# Electromagnetic Interference Shielding Properties of Highly Flexible Poly(styrene-co-butyl acrylate)/PEDOT:PSS Films Fabricated by Latex Technology

**DOI:** 10.3390/polym16111565

**Published:** 2024-05-31

**Authors:** Seung Chang Lee, Yong Bin Bang, Hyun Ho Park, Hyo Yeol Na, Seong Jae Lee

**Affiliations:** 1 Department of Polymer Engineering, The University of Suwon, 17 Wauan-gil, Bongdam-eup, Hwaseong 18323, Gyeonggi, Republic of Korea; 2 Department of Electronic Engineering, The University of Suwon, 17 Wauan-gil, Bongdam-eup, Hwaseong 18323, Gyeonggi, Republic of Korea; hhpark@suwon.ac.kr; 3 A2B2 Corp., 486 Chojeongyaksu-ro, Cheongwon-gu, Cheongju 28308, Chungbuk, Republic of Korea; hyna@a2b2.co.kr

**Keywords:** latex technology, electromagnetic interference shielding, flexible film, poly(styrene-co-butyl acrylate), PEDOT:PSS

## Abstract

As the use of stretchable electronic devices increases, the importance of flexible electromagnetic interference (EMI) shielding films is emerging. In this study, a highly flexible shielding film was fabricated using poly(styrene-co-butyl acrylate) (p(St-co-BA)) latex as a matrix and poly(3,4-ethylenedioxythiophene):poly(styrenesulfonate) (PEDOT:PSS) as a conductive filler, and then the mechanical properties and EMI shielding performance of the film were examined. Styrene and butyl acrylate were copolymerized to lower the high glass transition temperature and increase the ductility of brittle polystyrene. The latex blending technique was used to produce a shielding film in which the aqueous filler dispersion was uniformly dispersed in the emulsion polymerized resin. To determine the phase change in the copolymer matrix with temperature, the storage modulus was measured, and a time–temperature superposition master curve was constructed. The drying temperature of water-based copolymer resin suitable for film fabrication was set based on this curve. The glass transition temperature and flexibility of the blends were determined by evaluating the thermomechanical analysis and tensile tests. The EMI shielding effectiveness (SE) of the films was analyzed at frequencies from 50 MHz to 1.5 GHz, covering the VHF and UHF ranges. As the filler content increased, the SE of the blend film increased, but the elongation increased until a certain content and then decreased. The optimal content of PEDOT:PSS that satisfied both the ductility and shielding performance of the film was found to be 10 wt%. In this case, the elongation at break reached 300%, and the SE of a 1.6 mm thick film was about 35 dB. The film developed in this study can be used as an EMI shielding material that requires high flexibility.

## 1. Introduction

As scientific and technological progress continues, a vast array of electronic devices, including smartphones, portable computers, and wearable devices, are becoming increasingly ubiquitous. Portable devices that require miniaturization and lightweighting have increased electromagnetic interference (EMI) between adjacent parts within the device, so materials to shield it are certainly required [1,2,3,4]. Until now, they have been mainly used for electromagnetic wave shielding, but metal materials have disadvantages such as corrosiveness, low flexibility, and heaviness. To compensate for these shortcomings, the development of polymer-based shielding materials containing electrically conductive materials is actively being developed. Significant effects can be achieved by using conductive materials such as carbon nanotubes (CNTs), graphene, poly(3,4-ethylenedioxythiophene):poly(styrenesulfonate) (PEDOT:PSS), and silver nanowires [5,6,7,8,9,10,11]. In the authors’ previous research, an EMI shielding film was fabricated using CNT as a conductive nanofiller in a polymer matrix, and the shielding effect (SE) according to the filler content and film stacking was reported [5]. However, although CNTs with a large aspect ratio have high electrical conductivity, composite films composed of polymer and ceramic materials exhibit brittle properties due to the mixing of heterogeneous materials. In addition, CNTs have difficulty dispersing in aqueous phase, and to solve this problem, the surface needs to be modified with PSS, PEDOT:PSS, etc. Moreover, various difficulties occur, such as the surface of the CNTs not being partially modified, agglomeration of the CNTs occurring, or some parts not being uniformly dispersed within the matrix. An alternative to improve this problem may be to use the conductive polymer PEDOT:PSS directly as a filler in the polymer matrix, but it is also very difficult to uniformly disperse the filler surrounded by a shell of hydrophilic PSS in the hydrophobic matrix.

PEDOT:PSS is an electrically conductive polymer [12], and due to its unique properties, it exhibits excellent conductivity, thermal stability, and good water dispersibility when used as a filler [13,14,15,16,17]. Taking advantage of these various advantages, it is widely used as a commercially available conductive polymer and has recently been used to develop EMI shielding materials [18,19]. Additionally, when PEDOT:PSS is used as a filler when manufacturing films, a kind of plasticizer-like effect can be achieved [20]. Applying a certain amount of the organic additive PEDOT:PSS can, in most cases, produce conductive films with increased mechanical properties, especially elongation [21,22]. As reported in many previous studies, PEDOT:PSS can not only be used as a flexible thin film material but can also be used to manufacture flexible conductive films [23,24]. There are many studies on the mechanical properties of blend films containing PEDOT:PSS added to polymer resins [21,25,26,27,28]. In the case of using poly(vinyl alcohol) (PVA) and poly(ethylene oxide) (PEO) as polymer matrices, the mechanical properties differ depending on the type and molecular weight of PEO and PVA, which are soft polymers with relatively low elongation at break. It is known that when PEDOT:PSS is added, elongation increases as the content increases, but this decreases above a certain content [25].

Polystyrene (PS), a general-purpose polymer, is inexpensive and can be manufactured into films using various processing methods, but it has a high glass transition temperature (*T_g_*) and is brittle at room temperature [29], making it difficult to use as a flexible matrix. The copolymerization of styrene (St) and butylacrylate (BA) can lower the *T_g_* of the matrix material and provide flexibility even at room temperature [30]. Since poly(styrene-co-butyl acrylate) (p(St-co-BA)) is a copolymer latex prepared in the water phase, it has the advantage of dispersing with aqueous PEDOT:PSS dispersion. In addition, by analyzing the rheological properties of p(St-co-BA) depending on temperature, it is possible to understand the phase state and obtain information about optimal drying of the latex film [31,32]. In this study, a blend film with excellent elongation was successfully fabricated by adding a conductive polymer filler to an aqueous copolymer dispersion system using latex technology, and the optimal content that satisfies both flexibility and EMI SE was derived.

## 2. Experimental Section

### 2.1. Materials

**Scheme 1:**
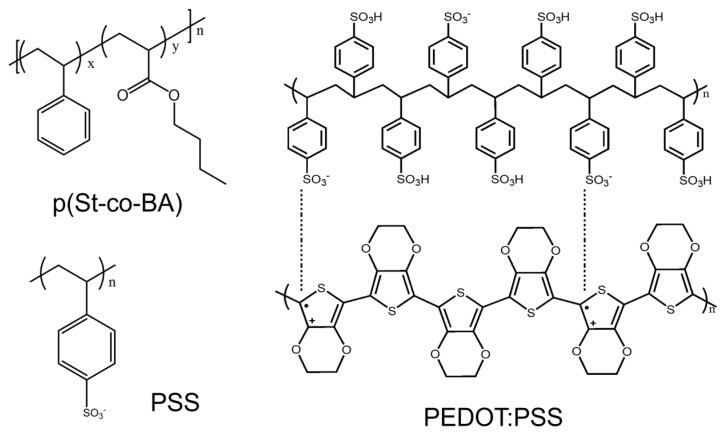
Chemical structures of p(St-co-BA), PSS, and PEDOT:PSS.

The two monomers St and BA, which are raw materials for the copolymer used as a matrix, were purchased from Samchun Chemical (Seoul, Republic of Korea) and used by distillation under reduced pressure. The initiator 2,2′-azobisisobutyronitrile (AIBN) used in the copolymerization process was also purchased from Samchun Chemical. Sodium dodecyl sulfate (SDS), an anionic surfactant, was purchased from Sigma-Aldrich (St. Louis, MO, USA). The conductive polymer PEDOT:PSS was provided by NanoChem Tech (Anseong, Republic of Korea). The electrical conductivity of the PEDOT:PSS is 1.3 S/cm according to the manufacturer’s information. Scheme 1 shows the chemical structures of p(St-co-BA), PSS, and PEDOT:PSS.

### 2.2. Preparation of p(St-co-BA) Latex

A total of 2 g of SDS was dissolved in 200 mL of deionized (DI) water, and 0.22 g of AIBN was added and stirred magnetically. In another beaker, 14.4 g of St and 8 g of BA were prepared by magnetic stirring. Then, the monomer mixture was added to the initiator aqueous solution, magnetically stirred for 1 h, and sonicated in an ultrasonic bath for 30 min. This mixture was copolymerized in a 500 mL three-neck double jacketed reactor under a nitrogen atmosphere at 90 °C for 2 h to synthesize p(St-co-BA) latex.

### 2.3. Fabrication of p(St-co-BA)/PEDOT:PSS Film

**Figure 1:**
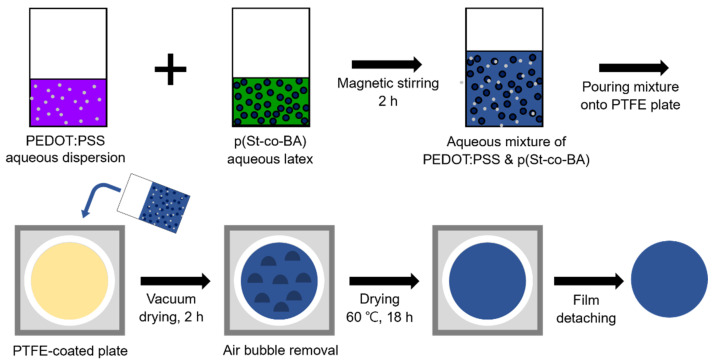
Schematic of p(St-co-BA)/PEDOT:PSS blend film fabrication.

In total, 1.4 wt% of PEDOT:PSS aqueous solution was added by content to 50 mL of DI water and magnetically stirred for 30 min. The p(St-co-BA) latex was added to this aqueous solution and magnetically stirred for another 2 h to prepare 4 g of an aqueous blend mixture based on the solid content. During mixing, the p(St-co-BA) latex particles could be well dispersed with the aqueous PEDOT:PSS dispersion. This was attributed to the negatively charged surface of the latex particle, resulting in electrostatic repulsion between the latex and filler particles. This mixture was poured onto a 15 cm (diameter) × 3 cm (height) circular mold coated with Teflon (PTFE), and air bubbles were removed using a vacuum oven at room temperature. Afterwards, it was dried in a convection oven at 60 °C for 18 h and then removed from the PTFE plate to prepare a 0.2 mm thick polymer blend film. Figure 1 is a schematic diagram of the process of manufacturing a blend film by dispersing PEDOT:PSS filler in a p(St-co-BA) matrix.

### 2.4. Characterization

The average diameter and size distribution of p(St-co-BA) latex particles were analyzed using a particle size analyzer (Nano ZS90, Malvern Panalytical, Worcestershire, UK). The morphology of p(St-co-BA) particles was analyzed using a field-emission scanning electron microscope (FE-SEM: Apreo, Thermo Fisher Scientific, Hillsboro, OR, USA) at a voltage of 10 kV and a magnification of ×100,000. Prior to SEM analysis, all samples were coated with gold. The *T_g_* of the copolymer was analyzed using a dynamic mechanical analyzer (DMA: DMA850, TA Instruments, New Castle, DE, USA). A specimen with a length of 13.7 mm, a width of 9.5 mm, and a thickness of 0.15 mm was prepared and analyzed at a heating rate of 3 °C/min over a temperature range of 0 to 70 °C at an amplitude of 15 μm and a frequency of 1 Hz. The rheological properties of p(St-co-BA) latex were analyzed using a rotational rheometer (MCR 302e, Anton Paar, Graz, Austria). The specimen for the measurement was prepared by compression molding into a disk-shaped specimen with a diameter of 25 mm and a height of 1 mm and was analyzed in parallel plate mode. The linear viscoelastic region was checked through a strain sweep in the range of 0.01% to 100% at a given temperature, and based on this, a frequency sweep was performed in the range of 0.03 to 100 rad/s. The mechanical properties were evaluated through a tensile test using a universal testing machine (UTM: LR10K Plus, Lloyd Instruments, West Sussex, UK) according to the ASTM D882 for thin plastic sheets. The elongation at break, elastic modulus, yield stress, and toughness were measured at a tensile speed of 1 mm/s at 25 °C for a rectangular film specimen with a length of 150 mm (grip distance of 100 mm), a width of 20 mm, and a thickness of 0.2 mm.

**Figure 2:**
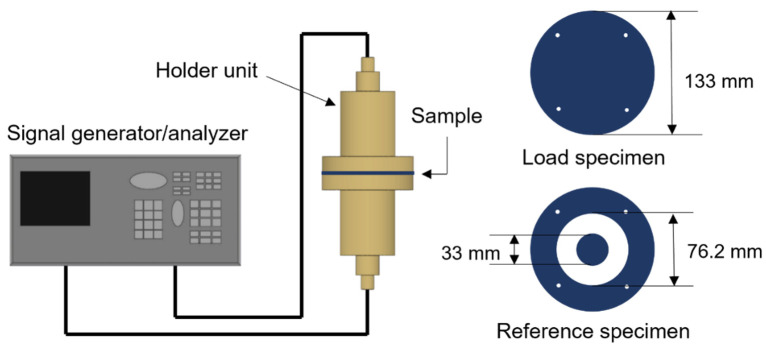
Schematic of EMI SE measuring system and specimen dimensions according to ASTM D4935 for planar materials.

EMI SE was analyzed using a network analyzer (E8358A, Agilent Technologies Inc., Santa Clara, CA, USA) according to the ASTM D4935 for planar materials. Figure 2 shows the EMI SE analysis equipment, measurement fixture, and dimensions of the reference and load specimens. The ASTM D4935 method measures two-port scattering parameters (*S*-parameters) using a network analyzer. To understand the EMI shielding characteristics of the film, the *S*-parameters (*S*_11_, *S*_22_, *S*_12_, and *S*_21_) expressed as the reflectance $R\;={\left|S_{11}\right|}^{2}={\left|S_{22}\right|}^{2}$, transmittance $T\;={\left|S_{12}\right|}^{2}={\left|S_{21}\right|}^{2}$, and absorbance A =1−R−T were analyzed [6,33]. The reflection shielding effectiveness (*SE_R_*), absorption shielding effectiveness (*SE_A_*), and total electromagnetic shielding effectiveness (*SE_T_*) can be obtained from the following Equations (1)–(3).

(1) $$SE_{R}=10\log\left(\frac{1}{1-R}\right)$$

(2) $$SE_{A}=10\log\left(\frac{1-R}{T}\right)$$

(3) $$SE_{T}=SE_{R}+SE_{A}$$

## 3. Results and Discussion

### 3.1. Morphology and Rheological Properties of p(St-co-BA) Copolymer

**Figure 3:**
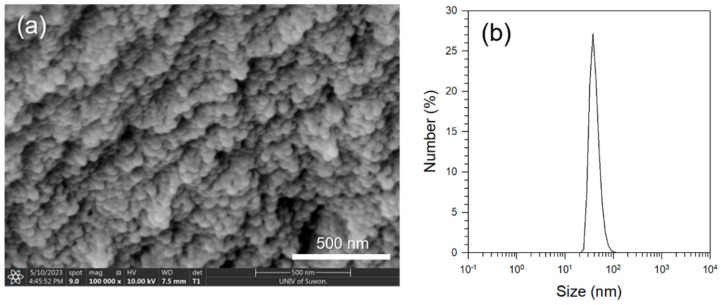
(**a**) SEM image of p(St-co-BA) latex particles at magnification of ×100,000 and (**b**) particle size distribution of p(St-co-BA) latex.

Copolymer p(St-co-BA) particles were observed using SEM, and their particle size distribution was analyzed using a particle size analyzer. The SEM image shown in Figure 3a revealed uniformly sized particles with a diameter of approximately 40–50 nm. The number average particle size evaluated from the particle size distribution in Figure 3b showed a very sharp peak at 41.9 nm. Through SEM and particle size analysis, it was confirmed that monodisperse copolymer particles with a size distribution of 30–50 nm (estimated at the half maximum) and an average diameter of 42 nm were obtained.

**Figure 4:**
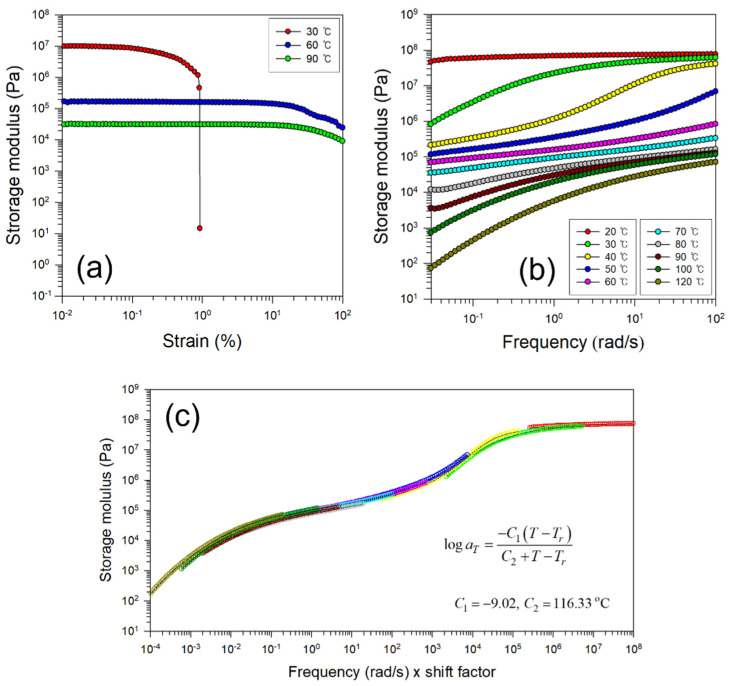
Rheological properties of p(St-co-BA): (**a**) strain sweep, (**b**) frequency sweep, and (**c**) time–temperature superposition master curve.

To determine the *T_g_* of the p(St-co-BA)/PEDOT:PSS polymer blend and the drying temperature suitable for film fabrication, the rheological properties of p(St-co-BA) were first measured. The linear viscoelastic range at a given temperature was established by performing a strain sweep (Figure 4a) from 0.01 to 100% at a frequency of 1 rad/s. As a result of strain sweep, the strains showing linear viscoelasticity at 30 °C, 60 °C, and 90 °C were determined to be 0.1%, 10%, and 10%, respectively. A frequency sweep was performed based on the strain at which linear viscoelasticity was maintained. Figure 4b shows the storage modulus (*G*′) measured from 20 °C to 120 °C. The plot of storage modulus vs. temperature showed glassy behavior with high *G*′ at low temperatures, and as the temperature increased, the behavior gradually changed to glass transition, rubber plateau, and rubbery/viscous flow. At a given temperature, *G*′ is found to increase with increasing frequency, because the polymer shows elastic-dominant behavior as the frequency increases. To understand the overall behavior of the copolymer from the glass phase to the melt phase, a master curve was constructed using the WLF equation (Equation (4)) based on the time–temperature superposition principle [34]. The modulus vs. frequency master curve shown in Figure 4c was constructed by shifting the modulus from a given frequency range obtained at various temperatures to a wide frequency range at a specific temperature.

(4) $$\log a_{T}=\frac{-C_{1}\left(T-T_{r}\right)}{C_{2}+T-T_{r}}$$

Here, the reference temperature *T_r_* was set to 70 °C. Through regression fitting from the shift factor *a_T_* for the temperature difference (*T* − *T_r_*) [35,36], the WLF constants *C*_1_ and *C*_2_ were obtained as −9.02 and 116.33 °C, respectively. It is clearly distinguished from the shape of the master curve of the copolymer that 20 °C is located in the glassy region, around 40 °C is in the glass transition region, and 60–70 °C is in the rubber plateau region. As the temperature further increases, the behavior gradually changes to the rubbery flow and viscous flow regions. Based on the shape of this master curve, the drying temperature of p(St-co-BA)/PEDOT:PSS film was estimated. In the glassy region, it takes a very long time to dry, and in the viscous flow region, it is difficult to maintain a uniformly dispersed state due to phase separation and sedimentation of the filler and matrix, especially at high temperatures. Drying in the rubber plateau region was therefore considered to be most appropriate and was set at 60 °C.

### 3.2. Thermomechanical Properties of p(St-co-BA)/PEDOT:PSS Blend

**Figure 5:**
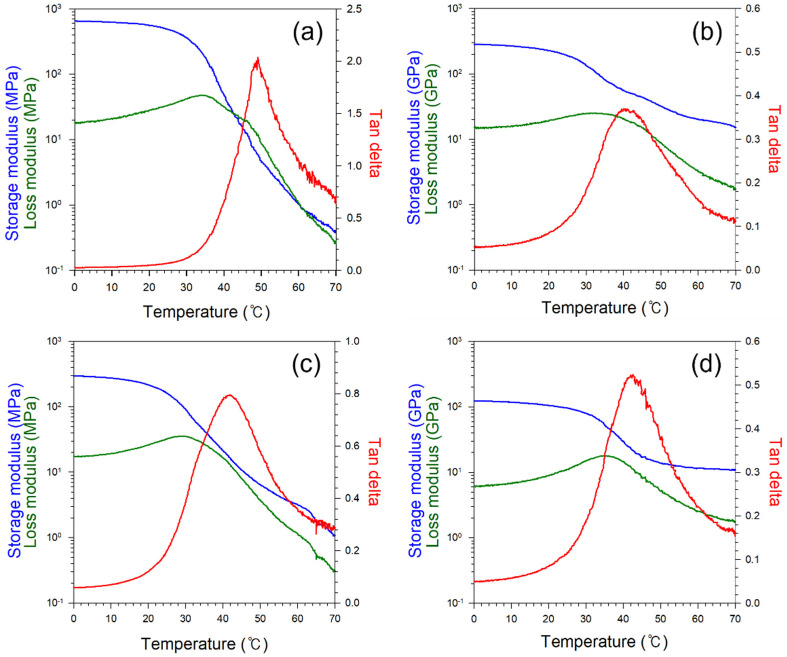
DMA thermograms of p(St-co-BA)/PEDOT:PSS films depending on PEDOT:PSS content: (**a**) 0 wt%, (**b**) 5 wt%, (**c**) 10 wt%, and (**d**) 15 wt%.

The dynamic mechanical properties of p(St-co-BA)/PEDOT:PSS films were measured to analyze *T_g_* changes with filler content. DMA thermograms such as storage modulus (*E*′), loss modulus (*E*”), and tan *δ* (=*E*”/*E*′) as a function of temperature in uniaxial tension mode are shown in Figure 5. The *T_g_* of a material can be obtained from the *E*′, *E*”, and tan *δ* graphs, respectively. The *T_g_* for each filler content was calculated from the temperatures at the *E*′ onset, *E*” peak, and tan δ peak, which are summarized in Table 1. Note that the *E*” peak occurs at a higher temperature than the *T_g_* measured through *E*′ onset and at a lower temperature than the tan *δ* peak, which is the usual case. As PEDOT:PSS was added to the p(St-co-BA) copolymer, *T_g_* decreased up to a 10 wt% addition of PEDOT:PSS and then tended to increase. Plasticization in blends of ionomers and soft polymers appears to need to consider both types of plasticization actions [30,37,38]. First, it is thought that the addition of PEDOT:PSS up to a certain amount acts as a plasticizer and reduces the interaction between copolymer chains, resulting in a lower *T_g_*. Second, the copolymer is also thought to lower the *T_g_* by weakening the interchain association of the ionic groups of PEDOT:PSS. However, when added above a certain amount, the phase separation of PEDOT:PSS seems to occur. It is inferred that the *T_g_* increases again due to the high *T_g_* (59 °C) caused by the strong interchain attraction between the rigid conjugate PEDOT and PSS in PEDOT:PSS [27,28].

### 3.3. Mechanical Properties of p(St-co-BA)/PEDOT:PSS Blend

**Figure 6:**
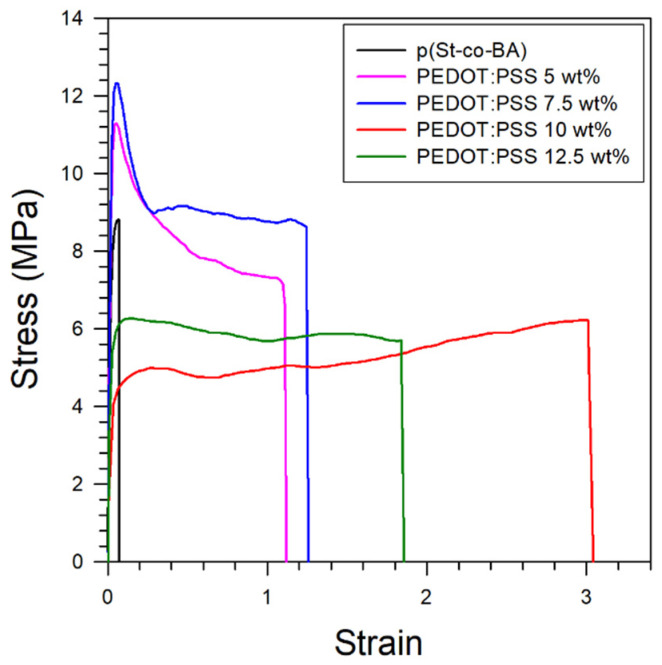
Stress–strain curves for PEDOT:PSS content in p(St-co-BA)/PEDOT:PSS films.

Figure 6 is a stress–strain graph evaluating the mechanical properties of the p(St-co-BA)/PEDOT:PSS blend at room temperature using a tensile test. Pristine p(St-co-BA) exhibited characteristics like a brittle polymer, showing a maximum strength at low elongation and then breaking immediately, i.e., tensile strength at break. The tensile strength and elongation at break of pristine PEDOT:PSS are known to be about 20 MPa and 5–8% [28]. The shape of the strain-stress curve of the blend of PEDOT:PSS added to the p(St-co-BA) matrix initially showed a clear yield strength and then plastic deformation, showing the behavior of typical soft polymers with very large elongations. As the PEDOT:PSS content increased up to 7.5 wt%, yield strength, elongation at break, and toughness all increased. At 10 wt%, the tensile modulus and yield strength decreased, and the elongation continued to increase, with a slight increase in tensile strength, showing behavior similar to the cold drawing phenomenon and then breaking. Table 2 summarizes the mechanical properties, such as tensile modulus, tensile strength at yield, elongation at break, and toughness from the stress–strain graph by filler content. The excellent elongation of this blend film is likely to be due to the similar small size of the p(St-co-BA) latex particles (~40 nm) and the water-dispersed PEDOTPSS particles (~30 nm [16]). The highest ductility was shown at a PEDOT:PSS content of around 10 wt%, and as the content was further increased, ductility tended to decrease and yield strength increased, as shown by the addition of 12.5 wt%. As discussed in the DMA analysis, it is understood that as the content of PEDOT:PSS was added up to 10 wt%, the interaction between molecules decreased and *T_g_* decreased, thereby increasing ductility. However, when added more than 10 wt%, it is inferred that *T_g_* increased again and flexibility decreased due to the phase separation of PEDOT:PSS and limited elongation at break (5–8% due to rigid PEDOT chains).

### 3.4. EMI Shielding Performance of p(St-co-BA)/PEDOT:PSS Blend Film

**Figure 7:**
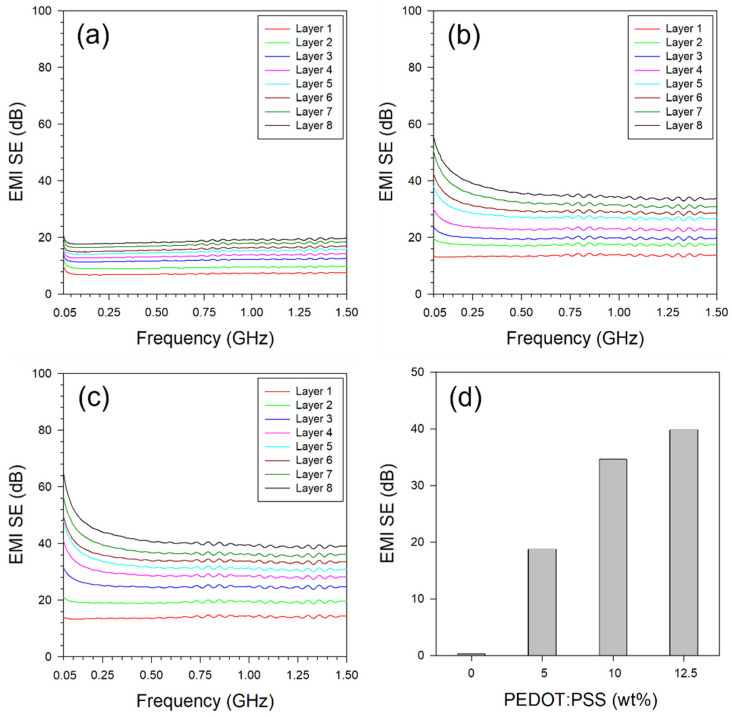
EMI shielding properties of p(St-BA)/PEDOT:PSS films by PEDOT:PSS content: (**a**) 5 wt%, (**b**) 10 wt%, (**c**) 12.5 wt%, and (**d**) comparison by content evaluated at 0.75 GHz.

The EMI shielding performance of p(St-co-BA) film and p(St-co-BA)/PEDOT:PSS films was analyzed. Figure 7 shows the SE measured by sequentially stacking 1 to 8 films with a thickness of 0.2 mm over a frequency range of 50 MHz−1.5 GHz. The pristine p(St-co-BA) film without PEDOT:PSS showed little SE even when up to eight films were stacked due to the absence of an electrically conductive filler. The SE of blend films containing 5 wt%, 10 wt%, and 12.5 wt% of PEDOT:PSS (Figure 7a–c) increased with the increase in the filler content and number of film stacks. When comparing eight-layered films (total thickness of 1.6 mm) at 0.75 GHz (Figure 7d), SE tended to increase linearly as the PEDOT:PSS content increased up to 12.5 wt%. Increased SE can be expected when using films with a high filler content, but it is difficult to manufacture a homogeneous blend. Additionally, due to its brittle nature, not only is it difficult to fabricate the film, but it is also difficult to prepare specimens for shielding analysis.

**Figure 8:**
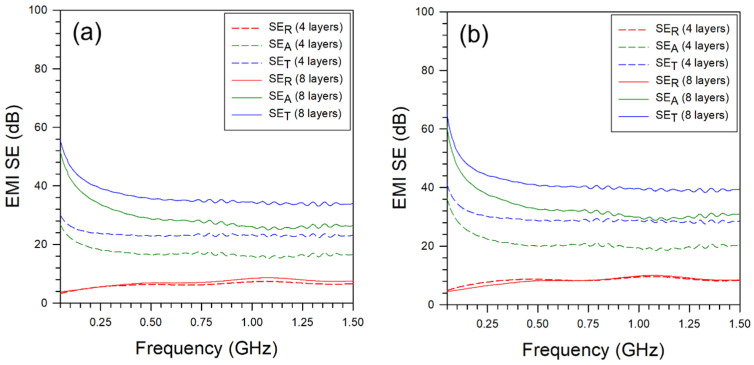
EMI shielding properties calculated by the measured S-parameters for p(St-BA)/PEDOT:PSS films with 4 and 8 layers: (**a**) PEDOT:PSS 10 wt% and (**b**) PEDOT:PSS 12.5 wt%.

Figure 8 shows graphs showing the *SE_A_*, *SE_R_*, and *SE_T_* of the p(St-co-BA)/PEDOT:PSS blend film with PEDOT:PSS content of 10 wt% and 12.5 wt%. In both films, *SE_A_* appeared to be greater than *SE_R_*, which is consistent with other studies showing that absorption shielding is the main mechanism for polymeric materials [39]. Additionally, as the thickness of the film increased, *SE_A_* increased, while *SE_R_* remained virtually unchanged. It is surmised that in reflection shielding, SE due to multiple reflections occurring inside the film is not significant.

## 4. Conclusions

A polymer blend film composed of PEDOT:PSS, a conductive filler surrounded by hydrophilic chain, and p(St-co-BA), a water-dispersed copolymer, was fabricated using latex technology. The p(St-co-BA), which has a *T_g_* of 30–50 °C, greatly improved the flexibility of brittle polystyrene. The rheological properties of p(St-co-BA) were measured to analyze *G*′ at various temperatures, and a master curve was constructed using the WLF equation. Through this curve, the phase change in the matrix by temperature was predicted, and the optimal drying temperature was set. The filler PEDOT:PSS had a similar effect as a plasticizer in a blend and resulted in a decrease in the *T_g_* of the film. This was also directly related to the mechanical properties. Up to 10 wt%, flexibility increased due to a decrease in *T_g_*, an increase in elongation at break, and a decrease in tensile modulus, but when the filler content exceeded this amount, *T_g_* increased, and the mechanical properties decreased. It appears to be an effect of the increased content of PEDOT’s rigid conjugated backbone. As the content of PEDOT:PSS increased, EMI SE steadily increased, but the mechanical properties rapidly decreased beyond a certain content. Therefore, it is most important to find the optimal content that can simultaneously satisfy the mechanical properties and EMI SE. The latex technology adopted in this study is a suitable method for manufacturing flexible EMI shielding films by achieving the homogeneous nanoscale dispersion of copolymer nanoparticle resin and conductive polymer nanofillers with controlled *T_g_* of the matrix.

## Figures and Tables

**Table 1:** Summary of *T_g_* of p(St-co-BA)/PEDOT:PSS films with various PEDOT:PSS contents.

Filler Content	0 wt%	5 wt%	10 wt%	15 wt%
*E*′ onset	31.9 °C	27.8 °C	24.9 °C	31.5 °C
*E*″ peak	35.3 °C	31.4 °C	29.5 °C	34.8 °C
tan *δ* peak	49.6 °C	41.9 °C	41.5 °C	44.6 °C

**Table 2:** Summary of tensile properties in p(St-co-BA)/PEDOT:PSS films.

PEDOT:PSS (wt%)	Young’s Modulus (MPa)	Tensile Strength at Yield (MPa)	Elongation at Break (%)	Toughness (MPa)
0 wt%	1720.3 ± 144.3	8.6 ± 0.7 *	4.5 ± 2.8	0.3 ± 0.2
5 wt%	506.6 ± 82.8	10.6 ± 0.7	81.1 ± 27.0	6.4 ± 2.4
7.5 wt%	427.5 ± 56.0	11.2 ± 1.4	116.6 ± 13.3	10.0 ± 1.5
10 wt%	121.0 ± 50.1	4.5 ± 0.5	305.4 ± 25.2	13.3 ± 2.7
12.5 wt%	248.4 ± 66.3	6.3 ± 0.6	161.1 ± 27.9	9.7 ± 1.7

* Since pristine p(St-co-BA) does not show a yield point, this value is the tensile strength at break.

## Data Availability

Data are contained within the article.
